# Phytoantioxidant Functionalized Nanoparticles: A Green Approach to Combat Nanoparticle-Induced Oxidative Stress

**DOI:** 10.1155/2021/3155962

**Published:** 2021-10-26

**Authors:** Acharya Balkrishna, Ashwani Kumar, Vedpriya Arya, Akansha Rohela, Rachna Verma, Eugenie Nepovimova, Ondrej Krejcar, Dinesh Kumar, Naveen Thakur, Kamil Kuca

**Affiliations:** ^1^Patanjali Herbal Research Department, Patanjali Research Institute, Haridwar 249405, India; ^2^Department of Allied Sciences, University of Patanjali, Haridwar 249405, India; ^3^School of Biological and Environmental Sciences, Shoolini University of Biotechnology and Management Sciences, Solan 173229, India; ^4^Department of Chemistry, Faculty of Science, University of Hradec Kralove, Hradec Kralove 50003, Czech Republic; ^5^Center for Basic and Applied Science, Faculty of Informatics and Management, University of Hradec Kralove, 50003 Hradec Kralove, Czech Republic; ^6^Malaysia Japan International Institute of Technology (MJIIT), Universiti Teknologi Malaysia, Jalan Sultan Yahya Petra, Kuala Lumpur 54100, Malaysia; ^7^School of Bioengineering and Food Technology, Shoolini University of Biotechnology and Management Sciences, Solan 173229, India; ^8^Department of Physics, Career Point University, Hamirpur 177001, India; ^9^Biomedical Research Center, University Hospital in Hradec Kralove, Sokolska 581, Hradec Kralove 50005, Czech Republic

## Abstract

Nanotechnology is gaining significant attention, with numerous biomedical applications. Silver in wound dressings, copper oxide and silver in antibacterial preparations, and zinc oxide nanoparticles as a food and cosmetic ingredient are common examples. However, adverse effects of nanoparticles in humans and the environment from extended exposure at varied concentrations have yet to be established. One of the drawbacks of employing nanoparticles is their tendency to cause oxidative stress, a significant public health concern with life-threatening consequences. Cardiovascular, renal, and respiratory problems and diabetes are among the oxidative stress-related disorders. In this context, phytoantioxidant functionalized nanoparticles could be a novel and effective alternative. In addition to performing their intended function, they can protect against oxidative damage. This review was designed by searching through various websites, books, and articles found in PubMed, Science Direct, and Google Scholar. To begin with, oxidative stress, its related diseases, and the mechanistic basis of oxidative damage caused by nanoparticles are discussed. One of the main mechanisms of action of nanoparticles was unearthed to be oxidative stress, which limits their use in humans. Secondly, the role of phytoantioxidant functionalized nanoparticles in oxidative damage prevention is critically discussed. The parameters for the characterization of nanoparticles were also discussed. The majority of silver, gold, iron, zinc oxide, and copper nanoparticles produced utilizing various plant extracts were active free radical scavengers. This potential is linked to several surface fabricated phytoconstituents, such as flavonoids and phenols. These phytoantioxidant functionalized nanoparticles could be a better alternative to nanoparticles prepared by other existing approaches.

## 1. Introduction

Nanotechnology is being designated the “next industrial revolution” since it is rapidly developing with the introduction of nanomaterial-based consumer goods [1, 2]. Nanoparticles (10^−9^m) are small objects that function as a whole unit in their transport and characteristics [3]. Nanoparticles (NPs) have diverse applications in disciplines like agriculture, healthcare, diagnosis, drug delivery, imaging, cosmetics, sunscreens, food, paints, catalysis, biolabeling, sensors, electronics, fiber optics, and other areas [2, 4–7]. Interestingly, most nanoparticles, such as Ag, Au, MgO, CuO, Al, CdS, and TiO_2_, are potent antibacterial agents [3, 8–10]. Copper oxide (CuO) NPs are used in antimicrobial products, cosmetics, heat transfer liquids, and semiconductors [11–13]. On the other hand, iron nanoparticles are used in biological material labeling and magnetic separation, drug delivery, and anticancer hyperthermia therapy [14]. Zinc oxide nanoparticles (ZnO NPs) are used in sunscreens, cosmetics, food additives, and packaging purposes [15–17]. Silver nanoparticles (Ag NPs) are popular due to their broad-spectrum antibacterial action [18–20]. Their therapeutic application ranged from silver-impregnated catheters to wound dressings [21, 22]. The silver-Acticoat™ dressing containing Ag NPs is superior to silver nitrate and silver sulfadiazine in wound healing [23, 24]. Ferric oxide (Fe_2_O_3_) NPs are used as catalysts and in the manufacture of pigments [25]. TiO_2_ NPs are believed to be the most valuable materials for cosmetics, food colorants, paper inks, pharmaceuticals, and protecting skin from UV rays [26–29]. Further, they have been used to prevent the spread of many infectious diseases [30, 31]. Furthermore, NPs including SiO_2_, TiO_2_, Bi_2_O_3_, Ag_2_O, FeO, MnO_2_, and Al_2_O_3_ play important roles in a variety of medicinal applications [32, 33]. In addition, AgS-, CuS-, FeS-, Zn-, and Cu-based metal organic frameworks are frequently utilized in drug delivery and antibacterial formulations [34].

Despite significant advances in nanomedicine, the long-term implications of NP exposure on human health and the environment are unknown [35]. When NPs enter the environment, they affect water, soil, and the air, where they might persist for a longer duration or be gobbled up by living organisms. They may biodegrade or bioaccumulate in the food chain, posing a hazardous risk [36–38]. The membrane injury, inflammatory response, DNA damage, and apoptosis have all been harmful consequences of ZnO NPs in mammalian cells [39–41]. Although Ag NPs are highly toxic to cancer cells, their use is limited since they are also hazardous to normal cells [35]. In continuation, when the toxicity of Ag NPs (10 *μ*g/mL) was investigated in human mesenchymal stem cells, DNA damage, impaired functioning, and cell death were observed [42]. Subsequently, ZnO NPs (300 mg/kg) caused oxidative stress in mice, which resulted in DNA damage [43]. Furthermore, intratracheal instillation of TiO_2_ NPs in mice resulted in accumulation of ROS, lipid peroxidation, and decreased antioxidant capacity [44]. Metal-derived NPs (copper, iron, cadmium, and silver) produce reactive oxygen species (ROS), which causes oxidative stress, restricting their wide-ranging application [45–50].

As evidenced by several pieces of research, oxidative stress is the key factor ascribed to the biological potential of nanoparticles [2, 35, 51]. ROS-induced oxidative stress destroys lipids, proteins, and DNA, and long-term exposure leads to neurological disorders, diabetes, rheumatoid arthritis, cancer, cardiovascular problems, and other diseases [52–56]. In the current situation, one interesting possibility for combating nanoparticle-induced oxidative stress could be the phytoantioxidant functionalized nanoparticles synthesized using plant extract-mediated green approach. Since these NPs contain a variety of surface-attached bioactive compounds from plants, they are referred to as phytoantioxidant functionalized NPs. The sonochemical, thermal decomposition, microwave aided, electrochemical, chemical reduction, and green synthesis are some of the chemical and physical methods used to synthesize NPs [57–62]. Unfortunately, many of these technologies employ hazardous chemicals, necessitate much energy, and produce poisonous by-products [63]. Green synthesis, which encompasses synthesis using plants, bacteria, fungus, algae, actinomycetes, and other organisms, is environmentally friendly, cost-effective, biocompatible, and safe [64–70]. Furthermore, phyto-mediated synthesis is preferable to a microbial method, which necessitates time-consuming and expensive downstream processing [71, 72].

Keeping in view the problem of nanoparticle-induced oxidative stress, in this review, we attempted to put some light on the antioxidant potential of phytoantioxidant functionalized NPs. Firstly, we have compiled a brief overview of oxidative stress-associated disorders and oxidative stress-mediated toxicity of nanoparticles. Secondly, a brief overview of green synthesis using plant extracts and the protective role of phytoantioxidant functionalized NPs as a therapeutic for oxidative stress is highlighted along with its mechanistic approaches.

## 2. Search and Inclusion Criteria

Science Direct, PubMed, and Google Scholar databases have been searched in this review with various keywords like oxidative stress, its related disorders, reactive oxygen and nitrogen species, mechanism of action, the toxicity of nanoparticles, oxidative stress-induced toxicity of nanoparticles, green synthesis, and antioxidant activity of nanoparticles. The literature review took place between May 1, 2021, and July 2, 2021. This review only considered full-length, original, and English-language papers from Web of Science, Scopus-indexed, and peer-reviewed journals.

## 3. Oxidative Stress and Its Related Disorders: A Brief Overview

Free radicals are strongly reactive atoms or molecules with unpaired electrons in their exterior shell and can be generated when oxygen reacts with specific molecules [73]. Free radicals, such as reactive oxygen species (ROS) and reactive nitrogen species (RNS), are produced continuously throughout cellular metabolism and play an important part in various cell signaling pathways [74–78]. Various endogenous and external activities generate ROS and RNS, and antioxidant defenses mitigate their harmful effects. Superoxide radicals (O_2_^·−^), hydroxyl for (^·^OH), hydrogen peroxide radicals (H_2_O_2_), and singlet oxygen are generally defined ROS [79–82]. RNS comprises nitric oxide, peroxynitrite (ONOO^−^), and their reaction products [83].

The protein phosphorylation, transcription factor activation, immunity, apoptosis, and differentiation are all reliant on adequate ROS production within cells, which must be maintained at a minimum level [84]. The ROS generation occurs mainly in the mitochondria, cell membranes, and cytoplasm. Even though these organelles have an inherent potential to scavenge ROS [85], it is noteworthy that this is insufficient to fulfill the cellular demand to eliminate the quantity of ROS generated by mitochondria [86].

**Figure 1:**
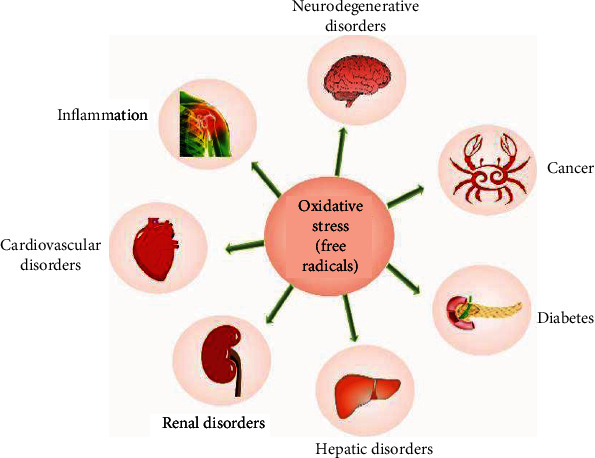
Various indications associated with the generation of oxidative stress.

The disparities between ROS and RNS generation and antioxidant defenses cause oxidative stress. Furthermore, when the formation of ROS rises, they begin to have adverse effects on essential cellular components (lipids, proteins, and nucleic acids) [77, 78, 87–89]. A substantial body of evidence suggests that oxidative stress has a role in the genesis and overall progress of various diseases, including diabetes; metabolic disorders; atherosclerosis; neurological, cardiovascular, and respiratory disorders; and cancer [90–92]. In Figure 1, several disorders linked to oxidative stress are presented.

Furthermore, as the level of antioxidant enzymes, glutathione peroxidase (GPx), catalase (CAT), and superoxide dismutase (SOD), diminishes with age, the age-related functional losses are attributed to ROS- and RNS-mediated lipid, protein, and DNA damage [93, 94]. Oxidative stress has a role in vascular endothelial dysfunction with age [95]. Several reports (both *in vivo* and *ex vivo*) shred the threads of evidence against oxidative stress-induced atherosclerosis, hypertension, ischemic heart disease, cardiomyopathy, and congestive heart failure [96–99]. Importantly, oxidative stress causes cardiovascular complications in type 2 diabetes (T2D) subjects by promoting prothrombotic reactions [100]. ROS are also associated with cardiac arrest by cardiac hypertrophy development, ischemia-reperfusion injury, and myocyte apoptosis [101–103].

There are reports that asthma and chronic obstructive pulmonary disease (COPD) are linked to ROS-induced oxidative stress [104–106]. Choudhury and MacNee [107] reported increased levels of oxidative stress biomarkers (8-hydroxydeoxyguanosine, protein carbonyl, 3-nitrotyrosine, F2-isoprostanes, and advanced glycation end products) in COPD patients. Subsequently, oxidative stress contributes to chronic kidney disease (CKD) pathogenesis via glomerular damage, renal ischemia, inflammation, and endothelial dysfunction [97, 108, 109]. In CKD patients, polymorphonuclear leukocytes (PMNs) and monocytes are activated, resulting in increased release of nicotinamide adenine dinucleotide phosphate (NADPH) oxidase and myeloperoxidase (MPO), which promotes the formation of ROS [110].

On the other hand, oxidative stress disrupts neuronal and cellular processes and has been related to several neurological illnesses, including amyotrophic lateral sclerosis, depression, amnesia, and Parkinson's and Alzheimer's disease [56, 111, 112]. The lipid membrane is one of the most impacted structures in the brain due to redox imbalance [113]. Moreover, due to higher glucose, insulin blood levels, fatty acids, and impaired glutathione synthesis, diabetes individuals have substantial ROS levels [114]. Increased production of ROS causes a mutation in an oncogene, leading to cancer [115]. ROS disrupt the Akt/PI3K/ERK cell signaling pathway, diminishing proapoptotic proteins while boosting antiapoptotic genes [116, 117]. Subsequently, ROS has been linked to acute liver injury pathogenesis for a long time [118]. Similarly, multiple reports have shown that oxidative stress has a role in the etiology of inflammatory disorders like rheumatoid arthritis [119–121].

## 4. Oxidative Stress-Mediated Toxicity of Nanoparticles

Nanotechnology has found applications in a range of fields, including the environment, energy, food, and medicine. Nanoparticles are employed in biomedical applications because they have several advantages over bulk materials, including a higher surface-to-volume ratio, improved magnetic characteristics, thermal stability, and improved optical and mechanical properties. Despite the widespread use of NPs stated in Introduction, there is still a lack of ample knowledge about NP-mediated toxicity. However, there are reports that NPs induce toxicity via increasing intracellular ROS levels. Nanoparticle toxicity due to oxidative stress is well documented, restricting their usage in human patients.

In this context, using carboxy-2′,7′-dichlorofluorescein diacetate (H_2_DCFDDA) assay, the function of oxidative stress in the toxicity of iron oxide NPs against murine macrophage (J774) cells was examined. It was shown that exposing cells to a higher concentration of NPs (500 *μ*g/mL) enhanced the generation of ROS, resulting in cellular damage and death [122]. Subsequently, when human microvascular endothelial cells were exposed to iron NPs, they showed an increase in permeability, ascribed to ROS generation. Furthermore, when cells are exposed to iron NPs, ROS is proven to be a significant factor in modulating Akt/GSK-3-mediated cell permeability [123].

Ahamed et al. [51] investigated the CuO NP-induced genotoxic reaction in human pulmonary epithelial cells (A549) via the p53 pathway. CuO NPs increased the cell cycle checkpoint protein p53 and the DNA damage repair proteins Rad51 and MSH2. In a dose-dependent manner, CuO NPs also triggered oxidative stress (10, 25, and 50 *μ*g/mL), as evidenced by glutathione, CAT, and SOD depletion and the stimulation of lipid peroxidation. These findings show that CuO NPs exerted genotoxicity in A549 cells, which could be due to oxidative stress. Likewise, using the Alamar blue assay, the cytotoxicity of CuO, silicon oxide, and ferric oxide NPs against human laryngeal epithelial cells (HEp-2) was examined. CuO exhibited cytotoxicity; however, HEp-2 cells were unaffected by silicon oxide or ferric oxide even at high doses (400 *μ*g/cm^2^). CuO-induced oxidative stress was suggested by a considerable rise in amount of 8-isoprostanes and the ratio of oxidized glutathione to total glutathione [124]. In human liver cells (HepG2), the apoptotic and genotoxic capacity of ZnO NPs was investigated. Their cellular toxicity was also examined at the molecular level. HepG2 viability was reduced on exposure to ZnO NPs (14-20 *μ*g/mL) for 12 h, and the cell death that occurred was apoptosis. They also triggered DNA damage, as demonstrated by an upsurge in formamidopyrimidine DNA glycosylase- (Fpg-) sensitive regions mediated by oxidative stress. ROS led to a decline in mitochondria membrane capacity and a rise in the Bax/Bcl2 ratio, resulting in a mitochondria-mediated apoptosis pathway [2].

Similarly, the impact of oxidative stress in the toxicity of ZnO NPs against human skin melanoma (A375) cells was studied. ZnO NPs were reported to cause oxidative stress, as evidenced by lipid peroxidation and the depletion of antioxidant enzymes. In cells exposed to the ZnO NPs, DNA damage was seen, which could be mediated by oxidative stress [125]. The occurrence of oxidative damage in lipids and proteins of MRC-5 human lung fibroblasts after exposure to Au NPs was investigated *in vitro* by Li et al. [126]. In addition, Au NP-treated cells produced considerably higher lipid hydroperoxides, indicating lipid peroxidation. Furthermore, oxidative damage was confirmed by verifying malondialdehyde (MDA) protein adducts using western blot study.

**Figure 2:**
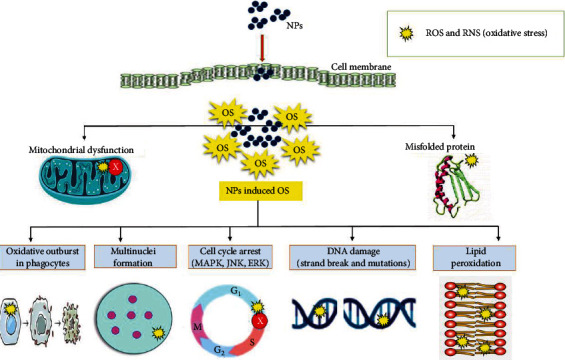
Mechanistic aspects of oxidative stress-mediated nanoparticle-induced toxicity. NPs: nanoparticles; ROS: reactive oxygen species; OS: oxidative stress; RNS: reactive nitrogen species.

The impact of oxidative stress on the cytotoxic and genotoxic potential of Ag NPs was investigated against human lung fibroblasts (IMR-90) and the human glioma (U251) cell lines. The findings revealed mitochondrial malfunction and ROS generation by Ag NPs, which resulted in DNA damage and chromosomal abnormalities. The mitochondrial respiratory chain disruption by Ag NPs is thought to cause the generation of ROS and the cessation of ATP synthesis, which leads to DNA damage [35]. Chairuangkitti et al. [127] evaluated the *in vitro* mechanisms of Ag NP (<100 nm) toxicity in connection to the ROS generation in A549 cells. Surprisingly, both ROS-dependent (cytotoxicity) and ROS-independent (cell cycle arrest) mechanisms are involved in Ag NP toxicity in A549 cells. The oxidative stress-dependent activity of NPs is depicted in Figure 2.

Several investigations using various human cells have added to our understanding of the underlying mechanism of NPs concerning ROS production. To a large extent, ROS formation causes cytotoxicity, genotoxicity, and signaling and inflammatory response activation, revealing the mutagenic and carcinogenic properties of NPs [51, 128, 129]. Increased ROS production is highly linked to the size and shape of NPs [130, 131]. However, because research reports vary, it is difficult to draw broad conclusions about shape and size.

In the event of NP exposure to cells, ROS generation is enhanced and leads to hyperoxidation of cell organelles, disruption of mitochondrial activity, endoplasmic reticulum (ER) stress, and unfolded protein response [129, 132–137]. Mitochondrial and ER stresses have cumulative effects on cell ROS production and apoptotic cell death, referred to as cytotoxicity [35, 132]. Furthermore, NPs in the nucleus cause oxidative base damages (8-oxoguanine), strand breakage, and mutations in DNA, resulting in genotoxicity [138–141]. Furthermore, NPs can mediate oxidative-sensitive activation of signaling cascades such as mitogen-activated protein kinase (MAPK), epidermal growth factor receptor, transcription factor activator protein-1, and nuclear factor-kappa B (NF-*κ*B), as well as activate the inflammatory response, which plays a role in mammalian growth and proliferative and developmental processes [97, 129, 142]. Subsequently, when phagocytes, such as neutrophils and macrophages, fail to phagocytose NPs completely, the NADPH-oxidase enzyme system produces ROS. The stimulation of cell signaling pathways such as MAPK, NF-*κ*B, Akt, and RTK by NP-induced ROS promotes an inflammatory cascade of chemokine and cytokine expression. The majority of the subsequent adverse effects elicited by NPs are due to ROS [129].

For instance, Chen and Schluesener [143] demonstrated that, to the human primary organ system, silver is relatively nontoxic and nonmutagenic. In contrast to antimicrobial metallic NPs (Au, Pt, Cu, Zn, Ti, and so on), silver is recognized to have the most potent antibacterial activity. Ag NPs' powerful antibacterial, antifungal, and antiviral properties are related to their potential to produce H_2_O_2_, O_2_^·−^, ^·^OH, and hypochlorous acid (HOCl) singlet oxygen [20, 144–147]. Furthermore, free radicals induced by Ag NPs reduce glutathione to glutathione disulfide that leads to oxidative stress, apoptosis, and stimulation of oxidative signaling pathways [51, 90, 128, 129, 148].

The cytotoxicity, genotoxicity, and inflammatory response of Ag NPs in cells have raised concerns about their unintended human exposure [149]. Ag NPs' cytotoxic, genotoxic, apoptotic, and antiproliferative effects, on the other hand, can be employed to treat glioblastoma [150, 151]. Dakal et al. [152] reported that Ag NP-induced ROS production and increased oxidative stress are linked to antimicrobial effects, with cytotoxic and genotoxic consequences. The most devastating and undeniable issue with using silver or any other nanoparticles in humans is their biosafety and biocompatibility.

## 5. Green Synthesis of NPs: A Brief Overview

**Figure 3:**
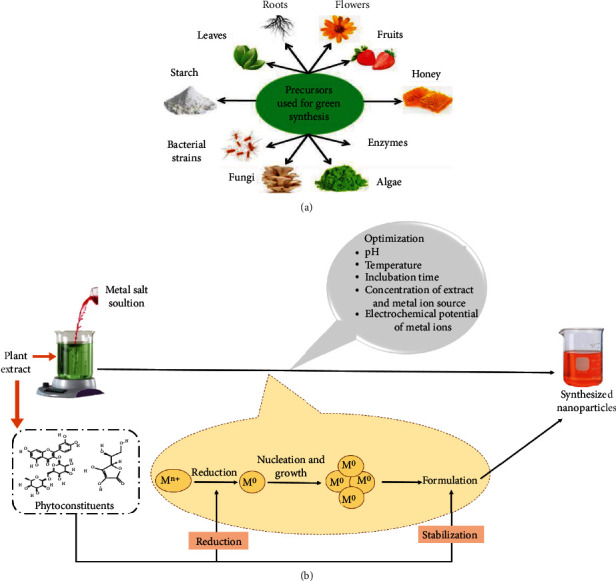
(a) Various precursors used for green synthesis of NPs; (b) mechanistic insight into plant-mediated green synthesis of metallic NPs. Reproduced from Kumar et al. [154] and Bhardwaj et al. [72] under Creative Commons Attribution (CC BY) license (http://creativecommons.org/licenses/by/4.0/).

Several methods for the synthesis of NPs have been developed, but their use in biomedical applications is limited due to the use of toxic compounds, the high energy requirements, and the formation of toxic by-products. The choice of a solvent medium, an environmentally friendly reducing agent, and a nontoxic substance for NP stabilization are all important components to consider during the NP preparation process [153]. In this context, green synthesis, which encompasses synthesis through plants, bacteria, fungi, algae, and others, is an effective way for generating NPs [64, 154] as shown in Figure 3(a). The ability of numerous biological entities, such as those indicated above, to generate metal nanoparticles for diverse pharmacological applications has been extensively researched. In general, plant extracts and microorganisms are used in the green, environmentally acceptable synthesis of NPs [65, 66].

Plant-derived NPs overwhelm microorganism-derived NPs, owing to the former's single-step, nonhazardous fabricating process [72]. Furthermore, phyto-mediated synthesis is preferable to microbial synthesis, which requires time-consuming and expensive downstream processing [71]. The plant extract is combined with a metal salt solution in the green synthesis of metal nanoparticles. The electrochemical potential of a metal ion and the pH of the reaction mixture, temperature, concentration, and reaction time are all critical aspects to consider. The phytoconstituents promote metal ion reduction to zero-valent state, followed by nucleation and growth to generate metal NPs [72, 154, 155] as depicted in Figure 3(b).

The plant-mediated synthesis is attributed to protein, phenols, terpenoids, ascorbic acid, and flavonoids that are capable of reducing the ions to nanosize and capping of nanoparticles [156, 157]. This method has several advantages, including energy savings due to the lack of high energy and pressure, use of biological entities that work as both reducing and stabilizing agents, environmental friendliness, lower costs, and the capacity to be employed on a large scale [64, 68, 69, 154]. Several NPs such as TiO_2_ using *Azadirachta indica* [9], ZnO using *Ocimum tenuiflorum*[158], CuO using *Ocimum tenuiflorum*[159], and ZnO using *Aloe vera*[160] have been successfully synthesized using the green approach.

## 6. Antioxidant Potential of Phytoantioxidant Functionalized Nanoparticles

Green synthesis is an innovative method of synthesizing phytoantioxidant functionalized NPs using plant extracts. It is gaining popularity as a result of its cost-effective, environmentally friendly, and large-scale production capabilities. As the importance of green synthesis using plant extracts is already highlighted in Section 5, in Table 1, the antioxidant potential of phytoantioxidant functionalized nanoparticles is shown. *Hibiscus rosa-sinensis* demonstrated excellent ability to synthesize copper NPs at optimal temperatures. These NPs showed good antioxidant potential in ferric-reducing antioxidant power (FRAP) and hydrogen peroxide (H_2_O_2_) assays [161]. Cu NPs synthesized using *Dioscorea bulbifera* tubers (DBTE) showed 40.81 ± 1.44, 79.06 ± 1.02, and 48.39 ± 1.46% scavenging against 1,1-diphenyl-2-picryl-hydrazyl (DPPH), nitric oxide (NO), and superoxide radicals (O_2_^·−^), respectively; this demonstrated its role in the prevention of oxidative stress, which is a significant factor in the progression of a variety of diseases. The action of DBTE is believed to be due to its substantial ascorbic acid content [162].

The aqueous fruit extract of *Couroupita guianensis* (CG), a promising bioreductant for reducing Au^3+^ ions into their nanoscale analogs, was used to produce gold nanoparticles (Au NPs) in a smaller duration of time. Au NPs have exceptional antioxidant characteristics; DPPH radical scavenging at 100 *μ*g/mL was 70.6%, compared to 96.28% for ascorbic acid. Further, they showed dose-dependent ferric ion reduction activity and hydroxyl (^·^OH) radical scavenging ability, which is primarily due to the presence of antioxidant residues, such as phenolics from the CG on its surface, which have been recognized, using various analytical techniques [67]. Subsequently, Au NPs were synthesized using *Rhus coriaria*, which is used as a reducing and capping agent. The plant polyphenols may play a role in reducing gold ions, as evident from FT-IR analysis. *In vitro*, antioxidant activity studies showed that DPPH (85.73% at 800 *μ*M) and ABTS activities (96.83% at 800 *μ*M) increased in a dose-dependent manner (25-800 *μ*M) which is related to the adsorption of phytocompounds on the surface of the Au NPs [163].

*Hippophae rhamnoides* leaves were utilized by Kalaiyarasan et al. [164] for the biosynthesis of silver NPs (Ag NPs). As the sample concentrations (5-25 *μ*g/mL) began to rise, the DPPH radical scavenging abilities increased, indicating that the antioxidant capabilities of the samples are dosage-dependent. The activity of Ag NPs has enhanced by more than tenfold when compared to that of the plant extract alone, which can be attributable to the presence of plant phytochemicals, including flavonoids. Due to these flavonoids and silver ions, antioxidant activity may occur via a single electron transfer mode [186, 187]. Similarly, Ag NPs developed using *Costus after* leaves were more effective DPPH scavengers than the leaf extract alone, and their antioxidant activity was comparable to those of ascorbic acid with IC_50_ value < 50 *μ*g/mL[165]. Ag NPs fabricated using *Taraxacum officinale* leaves demonstrated substantial antioxidant capability against ABTS (IC_50_ 45.6 *μ*g/mL), DPPH (IC_50_ 56.1 *μ*g/mL), and NO (IC_50_ 55.2 *μ*g/mL). Catechol and ascorbic acid were utilized as controls, with IC_50_ 40 to 60 *μ*g/mL [166]. Ag NPs synthesized using *Erythrina suberosa* leaves demonstrated significant antioxidant activity in a DPPH radical scavenging experiment, with IC_50_ 30.04 *μ*g/mL. The BHT was used as a standard. The findings significantly support the use of Ag NPs as natural antioxidants against oxidative stress-linked degenerative disorders [167].

Ag NPs synthesized using *Cestrum nocturnum* leaves, when evaluated for antioxidant activity, were found more active scavengers of DPPH (29.55% at 100 *μ*g/mL) as compared to ascorbic acid at a similar concentration (24.28%). Further, Ag NPs showed 45.41 and 20% scavenging of H_2_O_2_ and ^·^OH as compared to ascorbic acid (65.63 and 9.47% at 250 *μ*g/mL, respectively). However, negligible activity was reported in the O_2_^·−^ scavenging assay [168].

Subsequently, DPPH, H_2_O_2_, and FRAP assays were used to estimate the antioxidant activity of Ag NPs prepared using *Cassia angustifolia* flowers. Ag NPs were found to have FRAP and DPPH IC_50_ values of 63.21 ± 0.75 and 47.24 ± 0.5 *μ*g/mL, respectively. On the other hand, in the H_2_O_2_ assay, the IC_50_ value was 78.10 ± 1.2 *μ*g/mL [169]. The H_2_O_2_ scavenging is well related to the presence of phenolic components in the samples [188]. A compound mixture containing *Camellia sinensis* leaves, *Allium sativum* bulbs, and *Curcuma longa rhizome* mediated Ag NPs which were found highly active scavengers of ABTS, DPPH, ^·^OH, O_2_^·−^, and H_2_O_2_ radicals with IC_50_ ranging between 5.02 ± 1.11 and 22.93 ± 0.34 *μ*g/mL in comparison to rutoside and ascorbic acid (IC_50_ between 7.14 ± 1.02 and 14.17 ± 0.24 *μ*g/mL) [170]. Interestingly, Ag NPs synthesized using a spice blend exhibited IC_50_ < 31.25 and 68.75 *μ*g/mL against DPPH and ABTS, respectively, and the findings are comparable to standard rutoside. The different functional groups of spice blends present on the surface of Ag NPs could be responsible for the activity [171]. *Psidium guajava* leaves were utilized by Wang et al. [172] for the successful synthesis of Ag NPs, which were found to be highly active in scavenging free radicals with IC_50_52.53 ± 0.31 *μ*g/mL (DPPH) and 55.10 ± 0.29 *μ*g/mL (ABTS). In contrast, standard ascorbic acid showed IC_50_ < 40 *μ*g/mL against both DPPH and ABTS radicals. On the other hand, *Berberis aristata* leaf-mediated ZnO NPs, when evaluated for antioxidant activity using DPPH, showed 61.63% scavenging at 5 *μ*g/mL, lower than ascorbic acid (87.76%) at the same concentration [173]. On the other hand, Ag NPs synthesized using *Ananas comosus* fruit peel exhibited a moderate ABTS and DPPH and reduced power and nitric oxide (NO) scavenging activity as compared to standard BHT [174]. The action is due to the involvement of numerous plant functional groups attached to the surface of Ag NPs [189].

The antioxidant activity of Ag NPs fabricated using *Prosopis farcta* (PF) fruits was evaluated using DPPH and FRAP assay. Ag NPs had a scavenging activity of 43 to 63% at doses of 0.2-1 mg/mL; however, the effect was lower (74% at 1 mg/mL) than that of standard ascorbic acid. Ag NPs were also found active with FRAP activity > 25 mmol Fe(II)/mg extract at 1 mg/mL [175]. The primary phytochemicals responsible for the antioxidant capacity are phenolics and flavonoids, abundant in PF [190]. Subsequently, *Vitex negundo* leaf-mediated Au NPs exhibited IC_50_ values of 62.18 and 70.45 *μ*g/mL in DPPH and nitric oxide assays, respectively [176]. Moreover, *Morus alba* leaves were utilized by Das et al. [177] for synthesizing Ag NPs. When compared to ascorbic acid (10-50 *μ*g/mL), antioxidant activity of produced NPs against DPPH, ABTS, superoxide, and nitric oxide was dose-dependent with IC_50_ ranging between 25.9 and 97.2 *μ*g/mL. The lowest IC_50_ (25.9 *μ*g/mL) of Ag NPs was observed in the ABTS assay.

The DPPH radical scavenging activity of *Lavandula stoechas* aerial part-mediated Ag NPs (75% scavenging at 25 mg/mL) is attributed to phytochemical compounds such as phenol and terpenoid flavonoids that are involved in the reduction of Ag^+^ to Ag^0^[178]. *Nothapodytes foetida* leaf-mediated Ag NPs (100 *μ*g/mL) exhibited strong antioxidant potential with 93.80% inhibition of DPPH and comparatively lower ABTS radical scavenging activity (84.59% at the same conc.); however, results are comparable to standard BHT [179].

Likewise, Ansar et al. [180] utilized *Brassica oleracea* leaves for Ag NP synthesis. Ag NPs revealed a strong antioxidant potential against DPPH, NO, superoxide (O_2_^·−^), and hydroxyl radical (^·^OH) with percent scavenging ranged between 60 and 80% at 200 *μ*g/mL as compared to standard ascorbic acid (>80% at 200 *μ*g/mL). The antioxidant capacity of these nanoparticles could be attributed to the abundance of surface fabricated flavonoids and phenolics as capping agents [180]. In another study, Akintola et al. [181] investigated the antioxidant effects of Ag NPs (synthesized using *Blighia sapida* leaves) using DPPH and reducing power assay. Ag NPs at different concentrations (50, 75, 100, 125, and 150 *μ*g/mL) scavenged DPPH by 58.10, 59.26, 62.33, 71.24, and 75.42%, respectively. These effects, however, are less pronounced than those of ascorbic acid (>80% at 150 *μ*g/mL). Ag NPs had a maximum reduction capability of 53.52% at 150 *μ*g/mL compared to ascorbic acid (70.19%).

Similarly, iron NPs produced using *Asphodelus aestivus* aerial parts scavenged DPPH with IC_50_ 3.48 *μ*g/mL [182]. In addition, Rajput et al. [183] evaluated the antioxidant potential of Ag NPs (*Atropa acuminata* leaf mediated) using DPPH, H_2_O_2_, and O_2_^·−^ assay with IC_50_ 16.08, 25.4, and 21.12 *μ*g/mL, which is lower than standard ascorbic and gallic acid (IC_50_ 27.68-30.48 *μ*g/mL). In the DPPH and total antioxidant activity (TAA) assays, TiO_2_ NPs made from *Psidium guajava* leaves displayed strong antioxidant capability, with >85 and >90% scavenging at 500 *μ*g/mL, respectively. When compared to standard ascorbic acid, the action of NPs in TAA is more prominent [184]. On the other hand, different parts (leaves, pods, seeds, and seed shell) of *Cola nitida* (10-80 *μ*g/mL) exhibited 32.61-62.06% scavenging of DPPH. In addition, the scavenging ranged between 78.45 and 99.23% in H_2_O_2_ assay [185]. The antioxidant activity of phytoantioxidant functionalized NPs is related to the bioactive composition of the plant. The substantial body of research, including those selected in this study, did not go into extensive depth about plant selection. However, the selection of antioxidant-rich plants is the most important factor in the activity of phytoantioxidant functionalized NPs.

**Figure 4:**
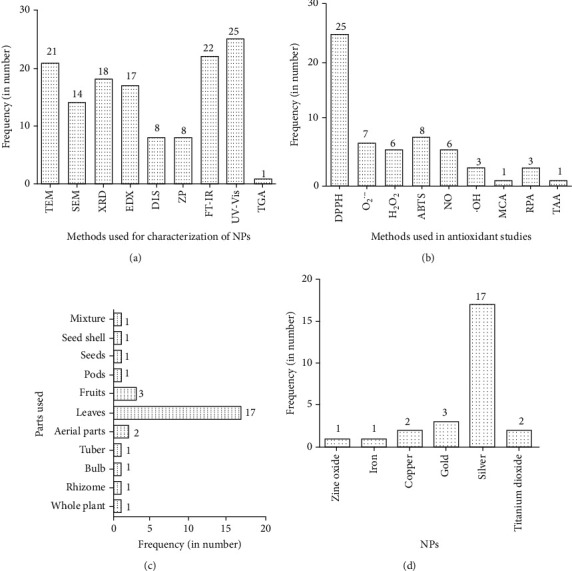
Frequency of methods used for (a) NP characterization, (b) antioxidant studies, (c) plant part used, and (d) types of NPs. Note: O_2_^·−^: superoxide radical; DPPH: DPPH radical scavenging activity; H_2_O_2_: hydrogen peroxide radical scavenging activity; ABTS: ABTS radical scavenging activity; ^·^OH: hydroxyl radical scavenging activity; NO: nitric oxide radical scavenging activity; FRAP: ferric-reducing antioxidant power; UV-Vis: ultraviolet and visible absorption spectroscopy; SEM: scanning electron microscopy; TEM: transmission electron microscopy; FT-IR: Fourier transform infrared spectroscopy; XRD: X-ray diffraction analysis; EDX: energy-dispersive X-ray spectroscopy; DLS: dynamic light scattering; TGA: thermal gravimetric analysis; TAA: total antioxidant activity.

The frequency of methodologies utilized for characterization of NPs, their significance, methods used for assessing antioxidant activity, plant parts used in green synthesis, and choice of NPs were all examined critically. The most commonly used strategy for antioxidant investigations was observed to be DPPH, followed by ABTS, superoxide, nitric oxide, and others. Surprisingly, all of the free radicals were successfully scavenged by the tested NPs. On the other hand, almost all plant parts have been used, but leaves are primarily harvested to synthesize NPs (Figure 4 and Table 1). Ag NPs are frequently employed in practice due to their inclusion in various formulations; the majority of researchers are currently focusing on these NPs, with silver topping the list of studies, followed by gold, copper, iron, and zinc oxide NPs.

The most common approach for measuring surface plasmon resonance and investigating the optical characteristics of produced NPs is UV-visible absorption spectroscopy, followed by FT-IR analysis to identify functional groups corresponding to surface-attached bioactive compounds responsible for reduction and stabilization. In 14, 21, and 18 investigations included in this study (*N* = 26), the shape and size of produced NPs were analyzed using scanning electron microscopy, transmission electron microscopy, and X-ray diffraction analysis. Dynamic light scattering analysis was also used to determine hydrodynamic size and surface charge. Zeta potential measurements were carried out to assess NP stability; a relatively high zeta potential value shows that the surface has a substantial electric charge, demonstrating its stability. The thermal stability of NPs was evaluated using thermal gravimetric analysis (TGA) in a single study (Figure 4 and Table 1). Furthermore, elemental analysis was carried out using energy-dispersive X-ray analysis (EDX), which is used to detect impurities [67, 163, 166, 169, 177, 182, 183].

## 7. Mechanistic Basis of Oxidative Stress Management by Phytoantioxidant Functionalized NPs

Antioxidant defenses mitigate the detrimental effects of ROS and RNS, which are generated by a variety of endogenous and external mechanisms [88]. NADPH oxidase, lipoxygenase, angiotensin II, and myeloperoxidase (MPO) are all endogenous producers of ROS and RNS [191]. NADPH oxidase produces superoxide radical (O_2_^·−^), which in turn is dissociated into the H_2_O_2_ by SOD [192]. Glutathione peroxidase (GPx), SOD, and CAT aid in the breakdown of free radicals into safe and less active molecules (H_2_O_2_/alcohol, and O_2_) [2, 193–198]. The phytoconstituents upregulate the level of antioxidant enzymes, and SOD is a significant force in radical neutralization [198]. As previously stated, SOD converts O_2_^·−^ to H_2_O_2_, which is then degraded by CAT and GPx into water and oxygen, preventing the formation of ^·^OH [199, 200]. Glutathione-S-transferase and glucose-6-phosphate dehydrogenase are two other antioxidant enzymes [200]. The inhibition of ^·^OH generation contributed to lipid radical (LR∗) inhibition, which is generated due to the interplay between ^·^OH and lipid membrane [198, 199]. GPx inhibits the peroxynitrite anion produced by numerous interactions, such as when combined with carbon dioxide to make nitrosoperoxycarbonate, which disintegrates over time to form nitrogen dioxide and carbonate radicals [198, 201].

**Figure 5:**
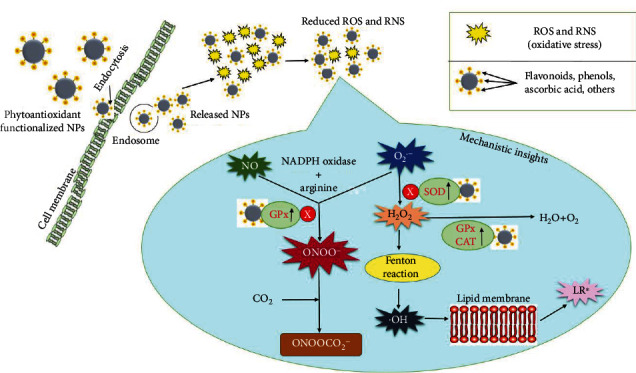
Role of phytoantioxidant functionalized nanoparticles in ameliorating oxidative stress. Note: GPx: glutathione peroxidase; CAT: catalase; O_2_^·−^: superoxide radical; H_2_O_2_: hydrogen peroxide; LR∗: lipid radical; H_2_O: water; O_2_: oxygen; SOD: superoxide dismutase; ^·^OH: hydroxyl radical; NO: nitric oxide; CO_2_: carbon dioxide; NADPH oxidase: nicotinamide adenine dinucleotide phosphate oxidase; ONOOCO_2_^−^: nitrosoperoxycarbonate; ONOO^−^: peroxynitrate.

On the other hand, nonenzymatic antioxidants interact with ROS and RNS to stop the free radical chain. In continuation, blood contains *α*-tocopherol, bilirubin, and *β*-carotene whereas albumin and uric acid make about 85% of antioxidant defense in plasma [89]. The phytoconstituents on nanoparticle surfaces reduce oxidative stress [72]. In Figure 5, a mechanistic approach to the protective impact of phytoantioxidant functionalized nanoparticles in the regulation of oxidative stress is highlighted.

The abundance of terpenoids, ascorbic acid, flavonoids, phenols, and other bioactive phytoconstituents on the surface of NPs is strongly correlated with their antioxidant activity [178, 180]. The phytoantioxidant functionalized nanoparticles would upregulate the antioxidant enzymes, and nonenzymatic components such as ascorbic acid on the surface of these NPs would also neutralize the adverse effects of free radicals.

## 8. Conclusion and Perspectives

In conclusion, the evidence of oxidative stress caused by nanoparticle exposure raises concerns about their use in humans. Although the antioxidant potential of phytoantioxidant functionalized NPs is well documented, the majority of the researches have been conducted *in vitro*. The bioactive substances like flavonoids and phenols are correlated to the antioxidant action of these NPs. The stability of nanoparticles is an important aspect, but only three articles have investigated the storage stability of these NPs. Most studies lack a zeta potential measurement, which is an indicator of stability. The data compiled in this review is expected to serve as a roadmap for researchers to fulfill various gaps. Because of their widespread use in a variety of industries, Ag NPs are the most investigated NPs. It is suggested that the plant utilized for green synthesis should be selected carefully, as antioxidant action is linked to phytoconstituents. As an alternative to NPs, phytoantioxidant functionalized NPs could be employed; however, high-quality toxicity studies are necessary. Nanotechnology is rapidly expanding, but research into the nanoparticles' toxicological impacts on human health and the environment is still in its early stages.

## Figures and Tables

**Table 1:** Characterization and antioxidant profile of phytoantioxidant functionalized nanoparticles.

Nanoparticle type	Plant (part used)	Reaction time (temp.)	Characterization methods	Size (nm)	Shape	Storage stability	Antioxidant assay and major findings (IC_50_*μ*g/mL)	Reference standard in antioxidant assay (IC_50_*μ*g/mL)	Data source
Copper	*Hibiscus rosa-sinensis* (leaves)	48 h (RT)	TEM, FT-IR, UV-Vis	NM	NM	n.d.	H_2_O_2_: 68.5% at 500 *μ*g/mL FRAP: OD: 1.2 at 1000 *μ*g/mL	DNS	[161]
Copper	*Dioscorea bulbifera* (tubers)	5 h (40°C)	TEM, EDX, XRD, DLS, FT-IR, UV-Vis	86-126	Spherical	n.d.	(% at 100 *μ*g/mL) DPPH: 40.81% NO: 79.06% O_2_^·−^: 48.39%	AA (100 *μ*g/mL) 51.42% 68.37% 14.11%	[162]
Gold	*Couroupita guianensis* (fruits)	1 h (70°C)	TEM, EDX, XRD, DLS, FT-IR, UV-Vis, zeta potential	26	Spherical, triangular, and hexagonal	45 days (RT)	(% and OD at 100 *μ*g/mL) DPPH: 70.6% O_2_^·−^: 89.8% Reducing power: OD: 0.3 ^·^OH (IC_50_): 36	AA 96.28% 90% OD: >0.7 <20 *μ*g/mL	[67]
Gold	*Rhus coriaria* (whole plant)	40 min (40°C) followed by 1 h (RT)	UV-Vis, XRD, TEM, FT-IR, zeta potential	15-25	Spherical	n.d.	(% at 800 *μ*M) DPPH: 85.73% ABTS: 96.83%	Glutathione (100%)	[163]
Silver	*Hippophae rhamnoides* (leaves)	24 h (RT)	TEM, UV-Vis, EDX, FT-IR, DLS, zeta potential	10-40	Spherical	1 year	DPPH: >80% at 20 *μ*g/mL	AA DNS	[164]
Silver	*Costus afer* (leaves)	2 h (90°C)	SEM, TEM, UV-Vis, EDX, FT-IR	20	Spherical	n.d.	DPPH (IC_50_): <50	Similar to AA	[165]
Silver	*Taraxacum officinale* (leaves)	15 min (RT)	TEM, XRD UV-Vis, FT-IR	5-30	Spherical	4 months (RT)	ABTS (IC_50_): 45.6 DPPH (IC_50_): 56.1 NO (IC_50_): 56.1	AA and catechol (IC_50_): 40-60	[166]
Silver	*Erythrina suberosa* (leaves)	Overnight (RT)	DLS, TEM, UV-Vis, FT-IR	12-115	Spherical	n.d.	DPPH (IC_50_): 30.04	BHT (DNS)	[167]
Silver	*Cestrum nocturnum* (leaves)	1 week (RT)	SEM, TEM, XRD, UV-Vis, FT-IR	20	Spherical	n.d.	DPPH: 29.55% H_2_O_2_: 45.41% ^·^OH: 20% O_2_^·−^: 8%	AA 24.28% 65.63% 9.47% 32%	[168]
Silver	*Cassia angustifolia* (flowers)	NM (27 ± 1°C)	UV-Vis, SEM, EDX, XRD, FT-IR, DLS, zeta potential	10-80	Spherical	n.d.	DPPH (IC_50_): 47.24 H_2_O_2_ (IC_50_): 78.10 FRAP (IC_50_): 63.21	AA (IC_50_) >60 in all tested assays	[169]
Silver	*Camellia sinensis* (leaves), *Allium sativum* (bulb), *Curcuma longa* (rhizome)	2 h (60°C)	SEM, TEM, UV-Vis, EDX, FT-IR	8	Spherical	n.d.	IC_50_ between 5.02 and 22.93 (ABTS, DPPH, ^·^OH, O_2_^·−^, H_2_O_2_)	AA and rutoside (IC_50_) 7.14 ± 1.02 to 14.17 ± 0.24	[170]
Silver	Spice blend	24 h (NM)	UV-Vis, EDX, SEM, XRD, TEM, FT-IR	6-28	Spherical	n.d.	DPPH (IC_50_): <31.2 ABTS (IC_50_): 68.75	Comparable to rutoside	[171]
Silver	*Psidium guajava* (leaves)	30 min (RT)	SEM, TEM, XRD, UV-Vis, EDX, FT-IR, zeta potential	20-35	Spherical	n.d.	DPPH (IC_50_): 52.53 ABTS (IC_50_): 55.10	AA (IC_50_) <40	[172]
Zinc oxide	*Berberis aristata* (leaves)	DNS	SEM, XRD, UV-Vis, FT-IR, EDX, DLS	5-40	Needle-like	n.d.	DPPH (IC_50_): 3.55	AA (IC_50_) 1.69	[173]
Silver	*Ananas comosus* (fruit peel)	20-30 min (100°C)	UV-Vis, SEM, EDX, XRD, FT-IR	NM	Spherical	n.d.	(% and OD at 100 *μ*g/mL) ABTS: 10-20% DPPH: <50% NO: <30% Reducing power: OD < 0.1	BHT (100 *μ*g/mL) >90% 80% >70% OD > 3.0	[174]
Silver	*Prosopis farcta* (fruits)	25-45 min (50-70°C)	UV-Vis, XRD, TEM	10-15	Spherical	n.d.	DPPH (IC_50_): 0.70 ± 0.08 FRAP: >25 mmol Fe(II)/mg extract	AA (IC_50_) (0.26 ± 0.09) NM	[175]
Gold	*Vitex negundo* (leaves)	Overnight (RT)	SEM, TEM, XRD, UV-Vis, FT-IR, EDX	20-70	Spherical	n.d.	DPPH (IC_50_): 62.18 NO (IC_50_): 70.45	DNS	[176]
Silver	*Morus alba* (leaves)	10 min (NM)	SEM, TEM, XRD, UV-Vis, EDX, DLS, FT-IR	12-39	Spherical	n.d.	IC_50_ between 25.9 and 97.2 (DPPH, ABTS, O_2_^·−^, NO, metal chelating)	AA (IC_50_) 10-50	[177]
Silver	*Lavandula stoechas* (aerial parts)	30 min (80°C)	UV-Vis, SEM, XRD, TEM, FT-IR	20-50	Spherical	n.d.	(% at 25 mg/mL) DPPH: 75%	AA (25 mg/mL) 100%	[178]
Silver	*Nothapodytes foetida* (leaves)	NM (80°C)	UV-Vis, TEM	20-50	Spherical	n.d.	DPPH (IC_50_): 22.56 ABTS (IC_50_): 41.47	Comparable to BHT	[179]
Silver	*Brassica oleracea* (leaves)	10 min (RT)	TEM, EDX, FT-IR, UV-Vis, zeta potential	20	Spherical	n.d.	(% at 200 *μ*g/mL) DPPH: 80% NO: 80% O_2_^·−^: 60-80% ^·^OH: 60-80%	AA (200 *μ*g/mL) >80% in all tested assays	[180]
Silver	*Blighia sapida* (leaves)	2 h (30 ± 2°C)	UV-Vis, FT-IR, SEM	50-70	Spherical	n.d.	(% at 150 *μ*g/mL) DPPH: 75.42% Reducing power: 53.52%	AA (150 *μ*g/mL) >80% 70.19%	[181]
Iron	*Asphodelus aestivus* (aerial parts)	20 min (50-60°C)	SEM, TEM, XRD, UV-Vis, FT-IR, EDX, TGA, zeta potential	20-25	NM	n.d.	DPPH (IC_50_): 3.48	NM	[182]
Silver	*Atropa acuminata* (leaves)	30 min (60°C)	TEM, XRD, EDX, DLS, zeta potential, UV-Vis, FT-IR	5-20	Spherical	n.d.	DPPH (IC_50_): 16.08 H_2_O_2_ (IC_50_): 25.4 O_2_^·−^ (IC_50_): 21.12	AA (IC_50_): 27.68 GA (IC_50_): 28.31 AA (IC_50_): 30.48	[183]
Titanium dioxide	*Psidium guajava* (leaves)	24 h (RT)	FT-IR, SEM, EDX, XRD	32.58	Spherical	n.d.	(% at 500 *μ*g/mL) DPPH: >85% TAA: >90%	AA (500 *μ*g/mL) >90% <50%	[184]
Titanium dioxide	*Cola nitida* (leaves, pods, seeds, and seed shell)	1 h (RT)	UV-Vis, FT-IR, TEM, XRD, EDX	25-191	Spherical	n.d.	(% at 10-80 *μ*g/mL) DPPH: 32.61-62.06% H_2_O_2_: 78.45-99.23 %	NM	[185]

Note: n.d.: not determined; NM: not mentioned; DNS: data not shown; AA: ascorbic acid; BHT: butylated hydroxytoluene; GA: gallic acid; IC_50_: half-maximal inhibitory concentration; %: percent scavenging; RT: room temperature; nm: nanometer; O_2_^·−^: superoxide radical; DPPH: DPPH radical scavenging activity; H_2_O_2_: hydrogen peroxide radical scavenging activity; ABTS: ABTS radical scavenging activity; ^·^OH: hydroxyl radical scavenging activity; NO: nitric oxide radical scavenging activity; FRAP: ferric-reducing antioxidant power; UV-Vis: ultraviolet and visible absorption spectroscopy; SEM: scanning electron microscopy; TEM: transmission electron microscopy; FT-IR: Fourier transform infrared spectroscopy; XRD: X-ray diffraction analysis; EDX: energy-dispersive X-ray spectroscopy; DLS: dynamic light scattering; TGA: thermal gravimetric analysis; TAA: total antioxidant activity.

## Data Availability

All data are included in the manuscript.

## Supplementary Materials

Supplementary Materials Graphical abstract.

## References

[1] Gerber C., Lang H. P. (2006). How the doors to the nanoworld were opened. *Nature Nanotechnology*.

[2] Sharma V., Anderson D., Dhawan A. (2012). Zinc oxide nanoparticles induce oxidative DNA damage and ROS-triggered mitochondria mediated apoptosis in human liver cells (HepG2). *Apoptosis*.

[3] Jeevanandam J., Barhoum A., Chan Y. S., Dufresne A., Danquah M. K. (2018). Review on nanoparticles and nanostructured materials: history, sources, toxicity and regulations. *Beilstein Journal of Nanotechnology*.

[4] Tan W. B., Jiang S., Zhang Y. (2007). Quantum-dot based nanoparticles for targeted silencing of HER2/neu gene via RNA interference. *Biomaterials*.

[5] Yoon K. Y., Hoon Byeon J., Park J. H., Hwang J. (2007). Susceptibility constants of Escherichia coli and Bacillus subtilis to silver and copper nanoparticles. *Science of the Total Environment*.

[6] Kreuter J., Gelperina S. (2008). Use of nanoparticles for cerebral cancer. *Tumori Journal*.

[7] Salam H. A., Rajiv P., Kamaraj M., Jagadeeswaran P., Gunalan S., Sivaraj R. (2012). Plants: green route for nanoparticle synthesis. *International Journal of Biological Sciences*.

[8] Kumar A., Singh S., Kumar D. (2014). Evaluation of antimicrobial potential of cadmium sulphide nanoparticles against bacterial pathogens. *International Journal of Pharmaceutical Sciences and Research*.

[9] Thakur B. K., Kumar A., Kumar D. (2019). Green synthesis of titanium dioxide nanoparticles using _Azadirachta indica_ leaf extract and evaluation of their antibacterial activity. *South African Journal of Botany*.

[10] Thakur N., Anu K. K., Kumar A., Kumar A. (2021). Effect of (Ag, Zn) co-doping on structural, optical and bactericidal properties of CuO nanoparticles synthesized by a microwave-assisted method. *Dalton Transactions*.

[11] Chang H., Jwo C. S., Lo C. H., Tsung T. T., Kao M. J., Lin H. M. (2005). Rheology of CuO nanoparticle suspension prepared by ASNSS. *Reviews on Advanced Materials Science*.

[12] Zhou K., Wang R., Xu B., Li Y. (2006). Synthesis, characterization and catalytic properties of CuO nanocrystals with various shapes. *Nanotechnology*.

[13] Aruoja V., Dubourguier H. C., Kasemets K., Kahru A. (2009). Toxicity of nanoparticles of CuO, ZnO and TiO_2_ to microalgae _Pseudokirchneriella subcapitata_. *Science of the Total Environment*.

[14] Huber D. L. (2005). Synthesis, properties, and applications of iron nanoparticles. *Small*.

[15] Gerloff K., Albrecht C., Boots A. W., Forster I., Schins R. P. F. (2009). Cytotoxicity and oxidative DNA damage by nanoparticles in human intestinal Caco-2 cells. *Nanotoxicology*.

[16] Jin T., Sun D., Su J. Y., Zhang H., Sue H. J. (2009). Antimicrobial efficacy of zinc oxide quantum dots against Listeria monocytogenes, Salmonella enteritidis, and Escherichia coli O157:H7. *Journal of Food Science*.

[17] Schilling K., Bradford B., Castelli D. (2010). Human safety review of nano titanium dioxide and zinc oxide. *Photochemical and Photobiological Sciences*.

[18] Lok C. N., Ho C. M., Chen R. (2006). Proteomic analysis of the mode of antibacterial action of silver nanoparticles. *Journal of Proteome Research*.

[19] Gogoi S. K., Gopinath P., Paul A. (2006). Green Fluorescent Protein-ExpressingEscherichiacolias a model system for investigating the antimicrobial activities of silver nanoparticles. *Langmuir*.

[20] Kim J. S., Kuk E., Yu K. N. (2007). Antimicrobial effects of silver nanoparticles. *Nanomedicine*.

[21] Samuel U., Guggenbichler J. P. (2004). Prevention of catheter-related infections: the potential of a new nano-silver impregnated catheter. *International Journal of Antimicrobial Agents*.

[22] Chen J., Han C. M., Lin X. W., Tang Z. J., Su S. J. (2006). Effect of silver nanoparticle dressing on second degree burn wound. *Zhonghua Wai Ke Za Zhi*.

[23] Dunn K., Edwards-Jones V. (2004). The role of Acticoat™ with nanocrystalline silver in the management of burns. *Burns*.

[24] Pasupuleti V. R., Prasad T. N. V., Shiekh R. A. (2013). Biogenic silver nanoparticles using Rhinacanthus nasutus leaf extract: synthesis, spectral analysis, and antimicrobial studies. *International Journal of Nanomedicine*.

[25] Montes-Hernandez G., Pironon J., Villieras F. (2006). Synthesis of a red iron oxide/montmorillonite pigment in a CO_2_-rich brine solution. *Journal of Colloid and Interface Science*.

[26] Hoffmann M. R., Martin S. T., Choi W. Y., Bahnemann D. W. (1995). Environmental applications of semiconductor photocatalysis. *Chemical Reviews*.

[27] Sinha A., Khare S. K. (2011). Mercury bioaccumulation and simultaneous nanoparticle synthesis by Enterobacter sp. cells. *Bioresource Technology*.

[28] Gélis C., Girard S., Mavon A., Delverdier M., Paillous N., Vicendo P. (2003). Assessment of the skin photoprotective capacities of an organo-mineral broad-spectrum sunblock on twoex vivoskin models. *Photodermatology Photoimmunology and Photomedicine*.

[29] Trouiller B., Reliene R., Westbrook A., Solaimani P., Schiestl R. H. (2009). Titanium dioxide nanoparticles induce DNA damage and genetic instability in vivo in mice. *Cancer Research*.

[30] Janson O., Gururaj S., Pujari-Palmer S. (2019). Titanium surface modification to enhance antibacterial and bioactive properties while retaining biocompatibility. *Materials Science and Engineering, C*.

[31] Mora C. L. C., Mueller A., Janssen A. P. G. Antibacterial medical product and method for producing same.

[32] Passagne I., Morille M., Rousset M., Pujalte I., L’Azou B. (2012). Implication of oxidative stress in size-dependent toxicity of silica nanoparticles in kidney cells. *Toxicology*.

[33] Yaqoob A. A., Ahmad H., Parveen T. (2020). Recent advances in metal decorated nanomaterials and their various biological applications: a review. *Frontiers in Chemistry*.

[34] Yaqoob A. A., Parveen T., Umar K., Ibrahim M. N. M. (2020). Role of nanomaterials in the treatment of wastewater: a review. *Water*.

[35] AshaRani P. V., Mun G. L. K., Hande M. P., Valiyaveettil S. (2009). Cytotoxicity and genotoxicity of silver nanoparticles in human cells. *ACS Nano*.

[36] Nowack B., Bucheli T. D. (2007). Occurrence, behavior and effects of nanoparticles in the environment. *Environmental Pollution*.

[37] Dhawan A., Sharma V. (2010). Toxicity assessment of nanomaterials: methods and challenges. *Analytical and Bioanalytical Chemistry*.

[38] Jamuna B. A., Ravishankar R. V., Kharisov B. I., Kharissova O. V., Rasika Dias H. V. (2014). Environmental risk, human health and toxic effects of nanoparticles. *Nanomaterials for Environmental Protection*.

[39] Gojova A., Guo B., Kota R. S., Rutledge J. C., Kennedy I. M., Barakat A. I. (2007). Induction of inflammation in vascular endothelial cells by metal oxide nanoparticles: effect of particle composition. *Environmental Health Perspectives*.

[40] Yang H., Liu C., Yang D., Zhang H., Xi Z. (2009). Comparative study of cytotoxicity, oxidative stress and genotoxicity induced by four typical nanomaterials: the role of particle size, shape and composition. *Journal of Applied Toxicology*.

[41] Osman I. F., Baumgartner A., Cemeli E., Fletcher J. N., Anderson D. (2010). Genotoxicity and cytotoxicity of zinc oxide and titanium dioxide in HEp-2 cells. *Nanomedicine*.

[42] Hackenberg S. A., Scherzed A., Kessler M. (2011). Silver nanoparticles: evaluation of DNA damage, toxicity and functional impairment in human mesenchymal stem cells. *Toxicology Letters*.

[43] Sharma V., Singh P., Pandey A. K., Dhawan A. (2012). Induction of oxidative stress, DNA damage and apoptosis in mouse liver after sub-acute oral exposure to zinc oxide nanoparticles. *Mutation Research*.

[44] Sun Q., Tan D., Ze Y. (2012). Pulmotoxicological effects caused by long-term titanium dioxide nanoparticles exposure in mice. *Journal of Hazardous Materials*.

[45] MacNee W., Donaldson K. (2003). Mechanism of lung injury caused by PM10 and ultrafine particles with special reference to COPD. *European Respiratory Journal*.

[46] Jia H. Y., Liu Y., Zhang X. J. (2009). Potential oxidative stress of gold nanoparticles by induced-NO releasing in serum. *Journal of the American Chemical Society*.

[47] Durocher S., Rezaee A., Hamm C., Rangan C., Mittler S., Mutus B. (2009). Disulfide-linked, gold nanoparticle based reagent for detecting small molecular weight thiols. *Journal of the American Chemical Society*.

[48] Kirchner C., Liedl T., Kudera S. (2005). Cytotoxicity of colloidal CdSe and CdSe/ZnS nanoparticles. *Nano Letters*.

[49] Moriwaki H., Osborne M. R., Phillips D. H. (2008). Effects of mixing metal ions on oxidative DNA damage mediated by a Fenton-type reduction. *Toxicology in Vitro*.

[50] Arora S., Jain J., Rajwade J. M., Paknikar K. M. (2008). Cellular responses induced by silver nanoparticles: _In vitro_ studies. *Toxicology Letters*.

[51] Ahamed M., Siddiqui M. A., Akhtar M. J., Ahmad I., Pant A. B., Alhadlaq H. A. (2010). Genotoxic potential of copper oxide nanoparticles in human lung epithelial cells. *Biochemical and Biophysical Research Communications*.

[52] He F., Zuo L. (2015). Redox roles of reactive oxygen species in cardiovascular diseases. *International Journal of Molecular Sciences*.

[53] Dias V., Junn E., Mouradian M. M. (2013). The role of oxidative stress in Parkinson’s disease. *Journal of Parkinson's Disease*.

[54] Zuo L., Zhou T., Pannell B. K., Ziegler A. C., Best T. M. (2015). Biological and physiological role of reactive oxygen species-the good, the bad and the ugly. *Acta Physiologica*.

[55] Tan B. L. (2015). Water extract of brewers’ rice induces apoptosis in human colorectal cancer cells via activation of caspase-3 and caspase-8 and downregulates the Wnt/*β*-catenin downstream signaling pathway in brewers’ rice-treated rats with azoxymethane-induced colon carcinogenesis. *BMC Complementary Medicine and Therapies*.

[56] Liu Z., Zhou T., Ziegler A. C., Dimitrion P., Zuo L. (2017). Oxidative stress in neurodegenerative diseases: from molecular mechanisms to clinical applications. *Oxidative Medicine and Cellular Longevity*.

[57] Rodriguez-Sanchez L., Blanco M. C., Lopez-Quintela M. A. (2000). Electrochemical synthesis of silver nanoparticles. *The Journal of Physical Chemistry B*.

[58] Mafuné F., Kohno J. Y., Takeda Y., Kondow T., Sawabe H. (2000). Structure and stability of silver nanoparticles in aqueous solution produced by laser ablation. *The Journal of Physical Chemistry B*.

[59] Salavati-Niasari M., Davar F., Mazaheri M., Shaterian M. (2008). Preparation of cobalt nanoparticles from [bis(salicylidene)cobalt(II)]-oleylamine complex by thermal decomposition. *Journal of Magnetism and Magnetic Materials*.

[60] Okitsu K., Ashokkumar M., Grieser F. (2005). Sonochemical synthesis of gold nanoparticles: effects of ultrasound frequency. *The Journal of Physical Chemistry B*.

[61] Gutiérrez-Wing C., Esparza R., Vargas-Hernández C., Fernández García M. E., José-Yacamán M. (2012). Microwave-assisted synthesis of gold nanoparticles self-assembled into self-supported superstructures. *Nanoscale*.

[62] Iravani S. (2011). Green synthesis of metal nanoparticles using plants. *Green Chemistry*.

[63] Arockiya Aarthi Rajathi F., Arumugam R., Saravanan S., Anantharaman P. (2014). Phytofabrication of gold nanoparticles assisted by leaves of _Suaeda monoica_ and its free radical scavenging property. *Journal of Photochemistry and Photobiology B: Biology*.

[64] Zahir A. A., Chauhan I. S., Bagavan A. (2014). Synthesis of nanoparticles using Euphorbia prostrata extract reveals a shift from apoptosis to G0/G1 arrest in Leishmania donovani. *Journal of Nanomedicine and Nanotechnology*.

[65] Kavitha K. S., Baker S., Rakshith D. (2013). Plants as green source towards synthesis of nanoparticles. *International Research Journal of Biological Sciences*.

[66] Akhtar M. S., Panwar J., Yun Y. S. (2013). Biogenic synthesis of metallic nanoparticles by plant extracts. *ACS Sustainable Chemistry and Engineering*.

[67] Sathishkumar G., Jha P. K., Vignesh V. (2016). Cannonball fruit (Couroupita guianensis, Aubl.) extract mediated synthesis of gold nanoparticles and evaluation of its antioxidant activity. *Journal of Molecular Liquids*.

[68] Singh J., Dutta T., Kim K. H., Rawat M., Samddar P., Kumar P. (2018). Green synthesis of metals and their oxide nanoparticles: applications for environmental remediation. *Journal of Nanobiotechnology*.

[69] Kumar Bachheti R., Fikadu A., Bachheti A., Husen A. (2020). Biogenic fabrication of nanomaterials from flower-based chemical compounds, characterization and their various applications: a review. *Saudi Journal of Biological Sciences*.

[70] Kumar H., Bhardwaj K., Dhanjal D. S. (2020). Fruit extract mediated green synthesis of metallic nanoparticles: a new avenue in pomology applications. *International Journal of Molecular Sciences*.

[71] Narayanan K. B., Sakthivel N. (2011). Green synthesis of biogenic metal nanoparticles by terrestrial and aquatic phototrophic and heterotrophic eukaryotes and biocompatible agents. *Advances in Colloid and Interface Science*.

[72] Bhardwaj K., Dhanjal D. S., Sharma A. (2020). Conifer-derived metallic nanoparticles: green synthesis and biological applications. *International Journal of Molecular Sciences*.

[73] Chandrasekaran A., Idelchik M. D. P. S., Melendez J. A. (2017). Redox control of senescence and age-related disease. *Redox Biology*.

[74] Liu Y., Imlay J. A. (2013). Cell death from antibiotics without the involvement of reactive oxygen species. *Science*.

[75] Sena L. A., Chandel N. S. (2012). Physiological roles of mitochondrial reactive oxygen species. *Molecular Cell*.

[76] Shadel G. S., Horvath T. L. (2015). Mitochondrial ROS signaling in organismal homeostasis. *Cell*.

[77] Smallwood M. J., Nissim A., Knight A. R., Whiteman M., Haigh R., Winyard P. G. (2018). Oxidative stress in autoimmune rheumatic diseases. *Free Radical Biology and Medicine*.

[78] Karagülle M., Kardeş S., Karagülle O. (2017). Effect of spa therapy with saline balneotherapy on oxidant/antioxidant status in patients with rheumatoid arthritis: a single-blind randomized controlled trial. *International Journal of Biometeorology*.

[79] Sato H., Shibata H., Shimizu T. (2013). Differential cellular localization of antioxidant enzymes in the trigeminal ganglion. *Neuroscience*.

[80] Navarro-Yepes J., Zavala-Flores L., Anandhan A. (2014). Antioxidant gene therapy against neuronal cell death. *Pharmacology and Therapeutics*.

[81] Keren I., Wu Y., Inocencio J., Mulcahy L. R., Lewis K. (2013). Killing by bactericidal antibiotics does not depend on reactive oxygen species. *Science*.

[82] Boonstra J., Post J. A. (2004). Molecular events associated with reactive oxygen species and cell cycle progression in mammalian cells. *Gene*.

[83] di Meo S., Reed T. T., Venditti P., Victor V. M. (2016). Role of ROS and RNS sources in physiological and pathological conditions. *Oxidative Medicine and Cellular Longevity*.

[84] Rajendran P., Nandakumar N., Rengarajan T. (2014). Antioxidants and human diseases. *Clinica Chimica Acta*.

[85] Hansen J. M., Go Y. M., Jones D. P. (2006). Nuclear and mitochondrial compartmentation of oxidative stress and redox signaling. *Annual Review of Pharmacology and Toxicology*.

[86] Glasauer A., Chandel N. S. (2014). Targeting antioxidants for cancer therapy. *Biochemical Pharmacology*.

[87] Lobo V., Patil A., Phatak A., Chandra N. (2010). Free radicals, antioxidants and functional foods: impact on human health. *Pharmacognosy Reviews*.

[88] Liguori I., Russo G., Curcio F. (2018). Oxidative stress, aging, and diseases. *Clinical Interventions in Aging*.

[89] Wu J. Q., Kosten T. R., Zhang X. Y. (2013). Free radicals, antioxidant defense systems, and schizophrenia. *Progress in Neuro-Psychopharmacology and Biological Psychiatry*.

[90] Rahman K. (2007). Studies on free radicals, antioxidants, and co-factors. *Clinical Interventions in Aging*.

[91] Taniyama Y., Griendling K. K. (2003). Reactive oxygen species in the vasculature. *Hypertension*.

[92] Pizzino G., Irrera N., Cucinotta M. (2017). Oxidative stress: harms and benefits for human health. *Oxidative Medicine and Cellular Longevity*.

[93] Abete P., Napoli C., Santoro G. (1999). Age-related decrease in cardiac tolerance to oxidative stress. *Journal of Molecular and Cellular Cardiology*.

[94] Beckman K. B., Ames B. N. (1998). The free radical theory of aging matures. *Physiological Reviews*.

[95] Donato A. J., Eskurza I., Silver A. E. (2007). Direct evidence of endothelial oxidative stress with aging in Humans. *Circulation Research*.

[96] Bahorun T., Soobrattee M. A., Luximon-Ramma V., Aruoma O. I. (2007). Free radicals and antioxidants in cardiovascular health and disease. *Internet Journal of Medical Update*.

[97] Droge W. (2002). Free radicals in the physiological control of cell function. *Physiological Reviews*.

[98] Chatterjee M., Saluja R., Kanneganti S., Chinta S., Dikshit M. (2007). Biochemical and molecular evaluation of neutrophil NOS in spontaneously hypertensive rats. *Cellular and Molecular Biology*.

[99] Ceriello A. (2008). Possible role of oxidative stress in the pathogenesis of hypertension. *Diabetes Care*.

[100] de Cristofaro R., Rocca B., Vitacolonna E. (2003). Lipid and protein oxidation contribute to a prothrombotic state in patients with type 2 diabetes mellitus. *Journal of Thrombosis and Haemostasis*.

[101] Sag C. M., Santos C. X., Shah A. M. (2014). Redox regulation of cardiac hypertrophy. *Journal of Molecular and Cellular Cardiology*.

[102] Zhou T., Prather E. R., Garrison D. E., Zuo L. (2018). Interplay between ROS and antioxidants during ischemia-reperfusion injuries in cardiac and skeletal muscle. *International Journal of Molecular Sciences*.

[103] Van der Pol A., Van Gilst W. H., Voors A. A., Van der Meer P. (2019). Treating oxidative stress in heart failure: past, present and future. *European Journal of Heart Failure*.

[104] Caramori G., Papi A. (2004). Oxidants and asthma. *Thorax*.

[105] Hoshino Y., Mishima M. (2008). Redox-based therapeutics for lung diseases. *Antioxidants and Redox Signaling*.

[106] Thimmulappa R. K., Chattopadhyay I., Rajasekaran S. (2020). Oxidative stress mechanisms in the pathogenesis of environmental lung diseases. *Oxidative Stress in Lung Diseases*.

[107] Choudhury G., MacNee W. (2017). Role of inflammation and oxidative stress in the pathology of ageing in COPD: potential therapeutic interventions. *Journal of Chronic Obstructive Pulmonary Disease*.

[108] Galle J. (2001). Oxidative stress in chronic renal failure. *Nephrology, Dialysis, Transplantation*.

[109] Balasubramanian S. (2013). Progression of chronic kidney disease: mechanisms and interventions in retardation. *Apollo Medicine*.

[110] Putri A. Y., Thaha M. (2014). Role of oxidative stress on chronic kidney disease progression. *Acta Medica Indonesiana*.

[111] Halliwell B. (2001). Role of free radicals in the neurodegenerative Diseases. *Drugs and Aging*.

[112] Singh R. P., Sharad S., Kapur S. (2004). Free radicals and oxidative stress in neurodegenerative diseases: relevance of dietary antioxidants. *Journal, Indian Academy of Clinical Medicine*.

[113] Rao A. V., Balachandran B. (2002). Role of oxidative stress and antioxidants in neurodegenerative diseases. *Nutritional Neuroscience*.

[114] Lee S. C., Chan J. C. (2015). Evidence for DNA damage as a biological link between diabetes and cancer. *Chinese Medical Journal*.

[115] Qian Q., Chen W., Cao Y. (2019). Targeting reactive oxygen species in cancer via Chinese herbal medicine. *Oxidative Medicine and Cellular Longevity*.

[116] Redza-Dutordoir M., Averill-Bates D. A. (2016). Activation of apoptosis signalling pathways by reactive oxygen species. *Biochimica et Biophysica Acta*.

[117] Mehta M., Dhanjal D. S., Paudel K. R. (2020). Cellular signalling pathways mediating the pathogenesis of chronic inflammatory respiratory diseases: an update. *Inflammopharmacology*.

[118] Ramachandran A., Jaeschke H. (2018). Oxidative stress and acute hepatic injury. *Current Opinion in Toxicology*.

[119] Quiñonez-Flores C. M., González-Chávez S. A., del Río Nájera D., Pacheco-Tena C. (2016). Oxidative stress relevance in the pathogenesis of the rheumatoid arthritis: a systematic review. *BioMed Research International*.

[120] Attia A. M. M., Ibrahim F. A. A., Abd el-Latif N. A. (2016). Therapeutic antioxidant and anti-inflammatory effects of laser acupuncture on patients with rheumatoid arthritis. *Lasers in Surgery and Medicine*.

[121] Jaswal S., Mehta H. C., Sood A. K., Kaur J. (2003). Antioxidant status in rheumatoid arthritis and role of antioxidant therapy. *Clinica Chimica Acta*.

[122] Naqvi S., Naqvi, Samim M. (2010). Concentration-dependent toxicity of iron oxide nanoparticles mediated by increased oxidative stress. *International Journal of Nanomedicine*.

[123] Apopa P. L., Qian Y., Shao R. (2009). Iron oxide nanoparticles induce human microvascular endothelial cell permeability through reactive oxygen species production and microtubule remodeling. *Particle and Fibre Toxicology*.

[124] Fahmy B., Cormier S. A. (2009). Copper oxide nanoparticles induce oxidative stress and cytotoxicity in airway epithelial cells. *Toxicology in Vitro*.

[125] Saud Alarifi D. A., Alkahtani S., Verma A., Ahamed M., Ahmed M., Alhadlaq H. A. (2013). Induction of oxidative stress, DNA damage, and apoptosis in a malignant human skin melanoma cell line after exposure to zinc oxide nanoparticles. *International Journal of Nanomedicine*.

[126] Li J. J., Hartono D., Ong C., Bay B., Yung L. L. (2010). Autophagy and oxidative stress associated with gold nanoparticles. *Biomaterials*.

[127] Chairuangkitti P., Lawanprasert S., Roytrakul S. (2013). Silver nanoparticles induce toxicity in A549 cells via ROS-dependent and ROS- independent pathways. *Toxicology in Vitro*.

[128] Johnston H. J., Hutchison G., Christensen F., Peters M., Hankin S., Stone S. (2010). A review of the in vivo and in vitro toxicity of silver and gold particulates: particle attributes and biological mechanisms responsible for the observed toxicity. *Critical Reviews in Toxicology*.

[129] Manke A., Wang L., Rojanasakul Y. (2013). Mechanisms of nanoparticle-induced oxidative stress and toxicity. *BioMed Research International*.

[130] Huang T., Holden J. A., Heath D. E., O'Brien-Simpson N. M., O'Connor A. J. (2019). Engineering highly effective antimicrobial selenium nanoparticles through control of particle size. *Nanoscale*.

[131] Cho S., Lee B., Park W., Huang X., Kim D. H. (2018). Photoperiodic flower mimicking metallic nanoparticles for image-guided medicine applications. *ACS Applied Materials and Interfaces*.

[132] Menu P., Mayor A., Zhou R. (2012). ER stress activates the NLRP3 inflammasome via an UPR-independent pathway. *Cell Death and Disease*.

[133] Akter M., Sikder M. T., Rahman M. M. (2018). A systematic review on silver nanoparticles-induced cytotoxicity: Physicochemical properties and perspectives. *Journal of Advanced Research*.

[134] Lee A. R., Lee S. J., Lee M. (2018). Editor’s highlight: a genome-wide screening of target genes against silver nanoparticles in fission yeast. *Toxicological Sciences*.

[135] Gaetke L. M., Kuang C. (2003). Copper toxicity, oxidative stress, and antioxidant nutrients. *Toxicology*.

[136] AshaRani P., Hande M. P., Valiyaveettil S. (2009). Anti-proliferative activity of silver nanoparticles. *BMC Cell Biology*.

[137] Zhang R., Piao M. J., Kim K. C. (2012). Endoplasmic reticulum stress signaling is involved in silver nanoparticles- induced apoptosis. *The International Journal of Biochemistry and Cell Biology*.

[138] Ahamed M., Karns M., Goodson M. (2008). DNA damage response to different surface chemistry of silver nanoparticles in mammalian cells. *Toxicology and Applied Pharmacology*.

[139] Foldbjerg R., Olesen P., Hougaard M., Dang D. A., Hoffmann H. J., Autrup H. (2009). PVP-coated silver nanoparticles and silver ions induce reactive oxygen species, apoptosis and necrosis in THP-1 monocytes. *Toxicology Letters*.

[140] Kim S., Choi J. E., Choi J. (2009). Oxidative stress-dependent toxicity of silver nanoparticles in human hepatoma cells. *Toxicology in Vitro*.

[141] Hudecová A., Kusznierewicz B., Rundén-Pran E. (2012). Silver nanoparticles induce premutagenic DNA oxidation that can be prevented by phytochemicals from Gentiana asclepiadea. *Mutagenesis*.

[142] Holmstrom K. M., Finkel T. (2014). Cellular mechanisms and physiological consequences of redox-dependent signalling. *Nature Reviews Molecular Cell Biology*.

[143] Chen X., Schluesener H. J. (2008). Nanosilver: a nanoproduct in medical application. *Toxicology and Applied Pharmacology*.

[144] Pellieux C., Dewilde A., Pierlot C., Aubry J. M. (2000). [18] Bactericidal and virucidal activities of singlet oxygen generated by thermolysis of naphthalene endoperoxides. *Methods in Enzymology*.

[145] Kim S. H., Lee H. S., Ryu D. S., Choi S. J., Lee D. S. (2011). Antibacterial activity of silver-nanoparticles against Staphylococcus aureus and Escherichia coli. *Korean Journal of Microbiology and Biotechnology*.

[146] Wu D., Fan W., Kishen A., Gutmann J. L., Fan B. (2014). Evaluation of the Antibacterial Efficacy of Silver Nanoparticles against _Enterococcus faecalis_ Biofilm. *Journal of Endodontics*.

[147] Kim J. Y., Sungeun K., Kim J., Jongchan L., Yoon J. (2005). The biocidal activity of nano-sized silver particles comparing with silver ion. *Journal of Korean Society of Environmental Engineers*.

[148] Fenoglio I., Corazzari I., Francia C., Bodoardo S., Fubini B. (2008). The oxidation of glutathione by cobalt/tungsten carbide contributes to hard metal-induced oxidative stress. *Free Radical Research*.

[149] Chopra I. (2007). The increasing use of silver-based products as antimicrobial agents: a useful development or a cause for concern. *Journal of Antimicrobial Chemotherapy*.

[150] Gopinath P., Gogoi S. K., Chattopadhyay A., Ghosh S. S. (2008). Implications of silver nanoparticle induced cell apoptosis forin vitrogene therapy. *Nanotechnolgy*.

[151] Urbańska K., Pająk B., Orzechowski A. (2015). The effect of silver nanoparticles (AgNPs) on proliferation and apoptosis of in ovo cultured glioblastoma multiforme (GBM) cells. *Nanoscale Research Letters*.

[152] Dakal T. C., Kumar A., Majumdar R. S., Yadav V. (2016). Mechanistic basis of antimicrobial actions of silver nanoparticles. *Frontiers in Microbiology*.

[153] Baruwati B., Varma R. S. (2009). High value products from waste: grape pomace Extract-A three-in-one package for the synthesis of metal nanoparticles. *ChemSusChem*.

[154] Kumar H., Bhardwaj K., Kuča K. (2020). Flower-based green synthesis of metallic nanoparticles: applications beyond fragrance. *Nanomaterials*.

[155] Kandiah M., Chandrasekaran K. N. (2021). Green synthesis of silver nanoparticles using Catharanthus roseus flower extracts and the determination of their antioxidant, antimicrobial, and photocatalytic activity. *Journal of Nanotechnology*.

[156] Kuppusamy P., Yusoff M., Maniam G., Govindan N. (2016). Biosynthesis of metallic nanoparticles using plant derivatives and their new avenues in pharmacological applications - An updated report. *Saudi Pharmaceutical Journal*.

[157] Jain S., Mehata M. S. (2017). Medicinal plant leaf extract and pure flavonoid mediated green synthesis of silver nanoparticles and their enhanced antibacterial property. *Scientific Reports*.

[158] Sharma S., Kumar K., Thakur N., Chauhan M. S. (2020). Ocimum tenuiflorum leaf extract as a green mediator for the synthesis of ZnO nanocapsules inactivating bacterial pathogens. *Chemical Papers*.

[159] Sharma S., Kumar K., Thakur N., Chauhan S., Chauhan M. S. (2021). Eco-friendly _Ocimum tenuiflorum_ green route synthesis of CuO nanoparticles: Characterizations on photocatalytic and antibacterial activities. *Journal of Environmental Chemical Engineering*.

[160] Sharma S., Kumar K., Thakur N., Chauhan S., Chauhan M. S. (2020). The effect of shape and size of ZnO nanoparticles on their antimicrobial and photocatalytic activities: a green approach. *Bulletin of Materials Science*.

[161] Subbaiya R., Selvam M. M. (2015). Green synthesis of copper nanoparticles from Hibicus rosasinensis and their antimicrobial, antioxidant activities. *Research Journal of Pharmaceutical, Biological and Chemical Sciences*.

[162] Ghosh S., More P., Nitnavare R. (2015). Antidiabetic and antioxidant properties of copper nanoparticles synthesized by medicinal plant Dioscorea bulbifera. *Journal of Nanomedicine and Nanotechnology*.

[163] Shabestarian H., Homayouni-Tabrizi M., Soltani M. (2017). Green synthesis of gold nanoparticles using sumac aqueous extract and their antioxidant activity. *Materials Research*.

[164] Kalaiyarasan T., Bharti V. K., Chaurasia O. P. (2017). One pot green preparation ofSeabuckthornsilver nanoparticles (SBT@AgNPs) featuring high stability and longevity, antibacterial, antioxidant potential: a nano disinfectant future perspective. *RSC Advances*.

[165] Elemike E. E., Fayemi O. E., Ekennia A. C., Onwudiwe D. C., Ebenso E. E. (2017). Silver nanoparticles mediated by Costus afer leaf Extract: Synthesis, Antibacterial, Antioxidant and electrochemical properties. *Molecules*.

[166] Saratale R. G., Benelli G., Kumar G., Kim D. S., Saratale G. D. (2018). Bio-fabrication of silver nanoparticles using the leaf extract of an ancient herbal medicine, dandelion (Taraxacum officinale), evaluation of their antioxidant, anticancer potential, and antimicrobial activity against phytopathogens. *Environmental Science and Pollution Research*.

[167] Mohanta Y. K., Panda S. K., Jayabalan R., Sharma N., Bastia A. K., Mohanta T. K. (2017). Antimicrobial, antioxidant and cytotoxic activity of silver nanoparticles synthesized by leaf extract of Erythrina suberosa (Roxb.). *Frontiers in Molecular Biosciences*.

[168] Keshari A. K., Srivastava R., Singh P., Yadav V. B., Nath G. (2020). Antioxidant and antibacterial activity of silver nanoparticles synthesized by _Cestrum nocturnum_. *Journal of Ayurveda and Integrative Medicine*.

[169] Bharathi D., Bhuvaneshwari V. (2019). Evaluation of the cytotoxic and antioxidant activity of phyto-synthesized silver nanoparticles using Cassia angustifolia flowers. *BioNanoScience*.

[170] Selvan D. A., Mahendiran D., Kumar R. S., Rahiman A. K. (2018). Garlic, green tea and turmeric extracts-mediated green synthesis of silver nanoparticles: Phytochemical, antioxidant and _in vitro_ cytotoxicity studies. *Journal of Photochemistry and Photobiology B: Biology*.

[171] Otunola G. A., Afolayan A. J. (2018). In vitroantibacterial, antioxidant and toxicity profile of silver nanoparticles green-synthesized and characterized from aqueous extract of a spice blend formulation. *Biotechnology and Biotechnological Equipment*.

[172] Wang L., Wu Y., Xie J., Wu S., Wu Z. (2018). Characterization, antioxidant and antimicrobial activities of green synthesized silver nanoparticles from Psidium guajava L. leaf aqueous extracts. *Materials Science and Engineering: C*.

[173] Chandra H., Patel D., Kumari P., Jangwan J. S., Yadav S. (2019). Phyto-mediated synthesis of zinc oxide nanoparticles of _Berberis aristata_ : Characterization, antioxidant activity and antibacterial activity with special reference to urinary tract pathogens. *Materials Science and Engineering C-Materials for Biological Applications*.

[174] Das G., Patra J. K., Debnath T., Ansari A., Shin H. S. (2019). Investigation of antioxidant, antibacterial, antidiabetic, and cytotoxicity potential of silver nanoparticles synthesized using the outer peel extract of Ananas comosus (L.). *PloS One*.

[175] Salari S., Bahabadi S. E., Samzadeh-Kermani A., Yosefzaei F. (2019). In-vitro evaluation of antioxidant and antibacterial potential of GreenSynthesized silver nanoparticles using Prosopis farcta fruit extract. *Iranian Journal of Pharmaceutical Research*.

[176] Veena S., Devasena T., Sathak S. S. M., Yasasve M., Vishal L. A. (2019). Green synthesis of gold nanoparticles from Vitex negundo leaf extract: characterization and in vitro evaluation of antioxidant-antibacterial activity. *Journal of Cluster Science*.

[177] Das D., Ghosh R., Mandal P. (2019). Biogenic synthesis of silver nanoparticles using S1 genotype of Morus alba leaf extract: characterization, antimicrobial and antioxidant potential assessment. *SN Applied Sciences*.

[178] Mahmoudi R., Aghaei S., Salehpour Z. (2020). Antibacterial and antioxidant properties of phyto-synthesized silver nanoparticles usingLavandula stoechasextract. *Applied Organometallic Chemistry*.

[179] Datkhile K. D., Patil S. R., Durgavale P. P., Patil M. N., Jagdale N. J., Deshmukh V. N. (2020). Studies on antioxidant and antimicrobial potential of biogenic silver nanoparticles synthesized using Nothapodytes foetida leaf extract (Wight) Sleumer. *Biomedical and Pharmacology Journal*.

[180] Ansar S., Tabassum H., Aladwan N. S. (2020). Eco friendly silver nanoparticles synthesis by _Brassica oleracea_ and its antibacterial, anticancer and antioxidant properties. *Scientific Reports*.

[181] Akintola A. O., Kehinde B. D., Ayoola P. B. (2020). Antioxidant properties of silver nanoparticles biosynthesized from methanolic leaf extract ofBlighia sapida. *In IOP Conference Series: Materials Science and Engineering*.

[182] Tuzun B. S., Fafal T., Tastan P. (2020). Structural characterization, antioxidant and cytotoxic effects of iron nanoparticles synthesized using Asphodelus aestivus Brot. aqueous extract. *Green Processing and Synthesis*.

[183] Rajput S., Kumar D., Agrawal V. (2020). Green synthesis of silver nanoparticles using Indian Belladonna extract and their potential antioxidant, anti-inflammatory, anticancer and larvicidal activities. *Plant Cell Reports*.

[184] Santhoshkumar T., Rahuman A. A., Jayaseelan C. (2014). Green synthesis of titanium dioxide nanoparticles using _Psidium guajava_ extract and its antibacterial and antioxidant properties. *Asian Pacific Journal of Tropical Medicine*.

[185] Akinola P. O., Lateef A., Asafa T. B., Beukes L. S., Hakeem A. S., Irshad H. M. (2020). Multifunctional titanium dioxide nanoparticles biofabricated via phytosynthetic route using extracts of _Cola nitida_ : antimicrobial, dye degradation, antioxidant and anticoagulant activities. *Heliyon*.

[186] Upadhyay N. K., Kumar M. S. Y., Gupta A. (2010). Antioxidant, cytoprotective and antibacterial effects of Sea buckthorn (_Hippophae rhamnoides_ L.) leaves. *Food and Chemical Toxicology*.

[187] Clarke G., Ting K. N., Wiart C., Fry J. (2013). High correlation of 2, 2-diphenyl-1-picrylhydrazyl (DPPH) radical scavenging, ferric reducing activity potential and total phenolics content indicates redundancy in use of all three assays to screen for antioxidant activity of extracts of plants from the Malaysian rainforest. *Antioxidants*.

[188] Gaddam S. A., Kotakadi V. S., Gopal D. S., Rao Y. S., Reddy A. V. (2014). Efficient and robust biofabrication of silver nanoparticles by Cassia alata leaf extract and their antimicrobial activity. *Journal of Nanostructure in Chemistry*.

[189] Adedapo A. A., Jimoh F. O., Afolayan A. J., Masika P. J. (2008). Antioxidant activities and phenolic contents of the methanol extracts of the stems of Acokanthera oppositifolia and Adenia gummifera. *BMC Complementary and Alternative Medicine*.

[190] Saumya S., Basha P. (2011). Antioxidant effect of Lagerstroemia speciosa Pers (Banaba) leaf extract in streptozotocin-induced diabetic mice. *Indian Journal of Experimental Biology*.

[191] Salisbury D., Bronas U. (2015). Reactive oxygen and nitrogen species: impact on endothelial dysfunction. *Nursing Research*.

[192] Genestra M. (2007). Oxyl radicals, redox-sensitive signalling cascades and antioxidants. *Cell Signal*.

[193] Dhalaria R., Verma R., Kumar D. (2020). Bioactive compounds of edible fruits with their anti-aging properties: a comprehensive review to prolong human life. *Antioxidants*.

[194] Nagmoti D. M., Khatri D. K., Juvekar P. R., Juvekar A. R. (2012). Antioxidant activity free radical-scavenging potential of Pithecellobium dulce Benth. seed extracts. *Free radical and Antioxidant*.

[195] Boora F., Chirisa E., Mukanganyama S. (2014). Evaluation of nitrite radical scavenging properties of selected Zimbabwean plant extracts and their phytoconstituents. *Journal of Food Processing*.

[196] Tehrani H. S., Moosavi-Movahedi A. A. (2018). Catalase and its mysteries. *Progress in Biophysics and Molecular Biology*.

[197] Rakotoarisoa M., Angelov B., Espinoza S., Khakurel K., Bizien T., Angelova A. (2019). Cubic liquid crystalline nanostructures involving catalase and curcumin: BioSAXS study and catalase peroxidatic function after cubosomal nanoparticle treatment of differentiated SH-SY5Y cells. *Molecules*.

[198] Balkrishna A., Rohela A., Kumar A. (2021). Mechanistic insight into antimicrobial and antioxidant potential of Jasminum species: a herbal approach for disease management. *Plants*.

[199] Wills E. D. (1971). Effects of lipid peroxidation on membrane-bound enzymes of the endoplasmic reticulum. *Biochemical Journal*.

[200] Birben E., Sahiner U. M., Sackesen C., Erzurum S., Kalayci O. (2012). Oxidative stress and antioxidant defense. *World Allergy Organization Journal*.

[201] Szabó C., Ischiropoulos H., Radi R. (2007). Peroxynitrite: biochemistry, pathophysiology and development of therapeutics. *Nature Reviews Drug Discovery*.

