# Supplementary figures and images for: Phytoantioxidant Functionalized Nanoparticles: A Green Approach to Combat Nanoparticle-Induced Oxidative Stress
Source: Oxid Med Cell Longev. 2021 Oct 26;2021:3155962. doi: 10.1155/2021/3155962 (PMC8563134; doi:10.1155/2021/3155962)

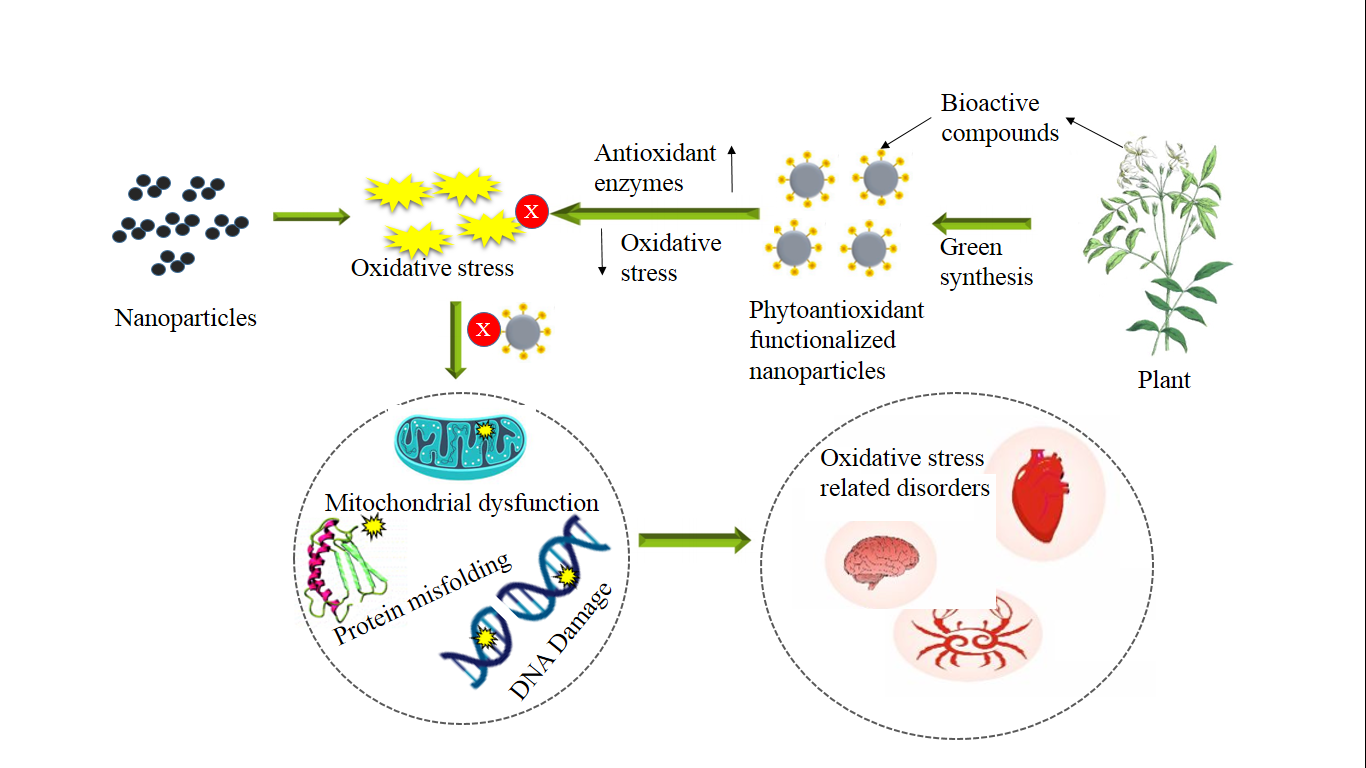

Supplement: Supplementary Materials — Graphical abstract. [file 3155962.f1.docx]
